# Educational Psychology Aspects of Learning with Chatbots without Artificial Intelligence: Suggestions for Designers

**DOI:** 10.3390/ejihpe13020022

**Published:** 2023-01-28

**Authors:** Michal Černý

**Affiliations:** Faculty of Art, Masaryk University in Brno, 602 00 Brno, Czech Republic; mcerny@phil.muni.cz

**Keywords:** chatbot, dialogue systems, information literacy, design through research, chatbot without AI, dialogue, e-learning, HCI

## Abstract

Chatbots without artificial intelligence can play the role of practical and easy-to-implement learning objects in e-learning environments, allowing a reduction in social or psychological isolation. This research, with a sample of 79 students, explores the principles that need to be followed in designing this kind of chatbot in education in order to ensure an acceptable outcome for students. Research has shown that students interacting with a chatbot without artificial intelligence expect similar psychological and communicative responses to those of a live human, project the characteristics of the chatbot from the dialogue, and are taken aback when the chatbot does not understand or cannot help them sufficiently. The study is based on a design through research approach, in which students in information studies and library science interacted with a specific chatbot focused on information retrieval, and recorded their experiences and feelings in an online questionnaire. The study intends to find principles for the design of chatbots without artificial intelligence so that students feel comfortable interacting with them.

## 1. Introduction

The COVID-19 pandemic has helped to identify many distance-education problems [1,2]. In agreement with other researchers, Adnan and Anwar [3] argue that students find it difficult to motivate themselves in online learning, they lack human contact, and that face-to-face interaction is essential to their learning. Bao [4], in one of the earliest studies, emphasised the importance of social connection and social interaction for learning. Thus, education stands at a crossroads where, on the one hand, there are legitimate demands for the development of online learning activities and programs [5,6], and, on the other hand, there are significant psychological [3,7] or psychosocial factors [8] that hinder students from making their learning sufficiently enjoyable and of high quality.

Online education is thus becoming both a challenge and a danger, facing the question of how to ensure the availability of quality education through digital technologies while supporting the social dimension of education, which is not to be neglected. There are several ways to tackle this challenge. The use of blended learning, which combines online and face-to-face learning, is offered [9,10]—the advantage of this is social interaction. The problem is that it requires people to come together, can lead to limiting the target group’s education, for example, geographically or socially. A combination of online and physical learning is offered by hybrid learning models [11], which are suitable for situations in which physical learning forms a substantial part of education and online participation opportunities include an integral complement. Another option may be synchronous online learning through online tutorials or webinars [12,13]. All of these solutions expect to educate individuals with sufficient time flexibility and language competence, although this represents a non-trivial exclusionary assumption.

Our study will focus on chatbots [14], which may represent a specific learning resource that will reduce the feeling of social isolation and promote the dimension of learning. The chatbot will not be seen as a substitute for a live teacher or tutor but rather as a complement to education that responds to the abovementioned challenges. In practice, we can encounter two basic types of chatbot: those that use artificial intelligence [15,16] and those that work with text-based frameworks for communication. The first group has the advantage of authenticity and the possibility of understanding and comprehending context, however, this places considerable demands on technical development and data is limited for so-called minor languages.

Chatbots based on text frames are cheap, easy to develop, and equally well-suited for small and large languages [17,18]. Their drawbacks are that the developer has to guess the direction of the dialogue accurately and that the chatbot has to be the one who actively manages the whole conversation. Abd-alrazaq et al. [19] report that most chatbots providing therapy in their research are deterministic algorithms, and only a minority use artificial intelligence. To develop a chatbot with AI, one needs to have a sufficiently large and high-quality training dataset for learning, which is problematic for minority topics and more minor languages. Developing a chatbot with AI is a purely professional job, primarily for a software engineer and their team. At the same time, a subject matter expert can create a chatbot based on deterministic decision-making. This makes the chatbot a standard educational resource easily modified by a particular teacher rather than by specialised software. Tamayo et al. [17] and Vázquez-Cano et al. [18] both highlight the close relationship between the sophistication of a chatbot’s psychological and pedagogical structure and its quality. Even a chatbot without AI can perform very well and can be usable for the educational process. Therefore, in our study, we find recommendations for developing chatbots as educational objects primarily by teachers, not as specialised software for one purpose.

Based on the qualitative data from the research we have conducted and on the literature, the aim of our study is to formulate recommendations for the developers of chatbots as specific learning objects, taking into account the pedagogical and psychological needs, and to measure the authenticity of the dialogue, user frustration, and the choice of linguistic resources. We will view chatbots as specific retrieval objects to help with social distance reduction, user motivation, active feedback, and other elements necessary for quality asynchronous distance learning.

### Chatbots

Brandtzaeg and Følstad [20] state that chatbots are software agents that serve as natural language user interfaces to provide data and services, nowadays usually for the needs of mobile devices. At the same time, they point out that the development of artificial intelligence carries an interest in this interaction. This definition is essential in highlighting that the chatbot is not the goal of action but rather a means of providing specific information. Dale [21] highlights the importance of dialogic–it is not one-way communication but dialogue, which, given Brandtzaeg and Følstad’s [20] definition, is usually quick and easy because it is done through short messages on a mobile phone. Jain et al. [22] refer to chatbots as conversational agents based on text messages. Indeed, it can be argued that the emphasis on dialogue and its interactivity in short text messages represents the most general model for understanding chatbots.

According to Reyes-Reina et al. [23], chatbots are characterised by four elements: (1) they simulate human speech; (2) they communicate via chat; (3) they have no physical image; and (4) they do not represent a human being in the virtual world. The first two points refer to the definition of the previous paragraph, and the last point distinguishes a chatbot from a chat device with a specific live person. The third component in this definition from Reyes-Reina et al. [23] is attractive, as it makes the chatbot a purely virtual entity–it is not a dialogical robot but a software agent.

Alan Turing was the first to develop the idea of human–computer dialogue using text input. His starting point was the belief that human thought is articulated through speech. Speech is the manifestation of thought. This belief, based on the ideas of analytic philosophy, was translated by Turing into the test named after him. The user has limited time to communicate textually through a computer with a particular entity and must decide whether they are speaking with a human or a software entity. According to Turing, if the software agent can have a dialogue that is indistinguishable from a human dialogue, it can be said to be thinking. Searle’s argument with the Chinese Room problem shows that the issue may be more profound, however, we believe that a counterexample needs to be convincing from Turing’s perspective. What is crucial is not “truth” per se, but rather how we as users experience interaction with the other.

In the 1960s, Josef Weizenbaum created the chatbot ELIZA to simulate a psychotherapeutic dialogue. The application worked on a straightforward principle: combining a search for keywords in the patient’s answers (and their translation into an interrogative form) with general questions that could be asked at almost any time in the “therapy”. Even though ELIZA did not possess anything that could be described as intelligence, it met the definition of a chatbot [22,23]. In the 1970s, the chatbot PARRY appeared, simulating a patient with schizophrenia. Its results were superior to those of ELIZA, although it did not need to express emotions, understand context, or work with context well. Its schizophrenia was a ploy to cover up its deficiencies in dialogue. In the late 1980s, Jabberwacky appeared as the first chatbot working with artificial intelligence. In 1995, ALICE appeared, inspired by ELZA but using web services and artificial intelligence. The modern understanding of chatbots is framed by voice and text assistants such as Siri, Google Assistant and IBM Watson.

Chatbots without artificial intelligence partly follow the tradition of ELIZA, in that they work with a clearly defined area of dialogue, which they determine based on interactions with the user. They cannot understand context or discussion. Their means of ensuring that the conversation is meaningful must be through well-posed questions and predicted responses to which the system has prepared answers in advance.

Chatbots are now being used in many fields: in marketing [24,25] healthcare [26], psychotherapy [27], in the industry for employee training [28], in banks [29], and in many other fields. 

We would like to specifically focus on the area of education [14,30]. Tamayo et al. [17] highlight the use of chatbots in areas such as:

- Intelligent tutoring systems [31];
- Improving student engagement [32];
- Intelligent feedback [33,34];
- Immediate assistance to the student [35];
- Teaching assistants [36,37,38];
- Mentors [39,40];
- Skills training [41].

All the publications listing the different areas of chatbot application we refer to here are only a small selection of the available literature from 2021–2022; this shows the relevance of chatbot use in education across a wide range of topics. Beyond Tamayo et al.’s list [17], specific chatbots emulating historical personhood through dialogue can be mentioned [42]. Neto and Fernandes [43] highlight the potential of using chatbots for collaborative learning and cooperation.

Kuhail et al. [44] report a division of chatbots according to educational approaches. This breakdown is significant because it shows different possible approaches to designing the chatbot as a learning object. Looking at the different studies that Kuhail et al. [44] work with, it is clear that multiple factors influence the choice of approach, however, the context or the specific educational problem that the chatbot is intended to solve is critical:

- Teaching agents use different teaching styles. They can refer to previous experiences. These chatbots aim to mimic the teacher’s work in his/her basic educational practices.
- Peer agents serve as tools to assist based on a student’s query, for example, in explaining a concept or working with a specific technology.
- Teachable agents try to teach students by systematically asking questions related to a specific problem. The student learns by seeking the answer to the questions step-by-step.
- Motivational agents work with thoughtful, emotional interaction design. Their goal may be to create a sense of empathy or encouragement, which can help students learn.

Mokmin and Ibrahim [45] highlight the importance of chatbots as a source of understanding specific aspects of health literacy, while also pointing out that a chatbot is highly motivating and leads to an understanding of issues relevant to college students. Essel et al. [46] work with an AI-free chatbot created without the need to code, and highlight that such a chatbot can serve as a pedagogical assistant, primarily if it works with motivating interactions for students. Topal et al. [47] emphasise working with a chatbot with artificial intelligence (formed in TezorFlow) for high school students, arguing that the fundamental impact is in the area of fun and that the chatbot positively impacts online learning. This research relied on data from COVID lessons where students found it challenging to feel isolated and alone. Gazulla et al. [48] highlight the need for students to participate in developing the chatbot that they are then to use. They work with the theme of self-directed learning, which the students themselves reflect on in creating the chatbot. Thus, participatory design leads to better outcomes in the form of the chatbot itself while at the same time developing students’ specific competencies and knowledge. Hew et al. [49] point out that poor student engagement in online learning is still problematic, and they see a chatbot acting as a friend of learners as one possible solution. Sriwisathiyakun and Dhamanitayakul [50] talk about the chatbot being an educational tool that has a natural feel and, at the same time, is associated with minimal barriers, allowing it to be used for competency-based learning. Furthermore, Kumar [51] highlights the potential of chatbots in competency learning and the social dimension of education. Abbas et al. [52] highlight the potential for using chatbots to reduce social barriers and to improve student engagement in the college community, especially for older and less traditional students.

Huang et al. [53] focus on the importance of chatbots in language learning while emphasising the need to develop chatbots iteratively, through systematic research with the target audience. It is possible to create a functional and robust learning object. A similar approach to iterative, incremental development (based on the wants and needs of the target group) can be seen in the work of Tamayo et al. [17] or Vázquez-Cano et al. [18]. Herrmann-Werner et al. [54] build on the above studies and emphasise the importance of reducing learner stress when working with a chatbot. Ranjan et al. [32] talk about the increase in learning efficiency, and Topal et al. [47] talk about the positive effect on students’ psyches. Thus, chatbots, when set up appropriately, can positively affect the overall educational climate.

## 2. Methodology

The topic of chatbots in the field of information education and librarianship is–unlike in fields such as mathematics education, languages or health literacy–very seldom reflected in chatbot research. Dube and Jacobs [55] write about using chatbots in academic libraries to improve orientation to the services offered. Mayr [56] discusses the possibility of implementing a chatbot as a reference librarian. Rodriguez and Mune [57] aim for some transition from a pure reference librarian to an educational instrument, although they do not emphasise information education. In this respect, our research design is unique. Thematically, only the studies of Lin et al. [58] are close, although they focus on the elderly. From their work, we also draw inspiration from the possibility of eliciting specific suggestions based on user interaction with the chatbot, along with a description of the system’s error rate.

In terms of formal methodology, this study could be classified as a case study (focusing on data from one focus group interacting with one particular chatbot) and an experiment. We follow the paradigmatic design through research approach. This is based on the idea that in developing a service, research can be conducted precisely when users interact with it and can reflect on this experience. This framework is also followed in our study.

In the third semester of their Bachelor’s degree, students in the field of Information Studies and Librarianship conducted chatbot testing as part of a compulsory course and recorded their reflections in Google Forms. In this way, we obtained 79 individual responses. Seventeen respondents were male, 54 were female, and one respondent identified as non-binary. Regarding age structure, 19 respondents were under the age of 20, 48 respondents were between 20–30, and 6 respondents were older.

Although the research uses a questionnaire, it is qualitatively oriented. We are not interested in obtaining information about the number of users but rather, given the nature of the topic, in forming principles for designers, i.e., in creating a new theoretical model. Therefore, we work qualitatively with the students’ free responses and choose open coding because we require an adequate theoretical framework for choosing codes. Individual statements are not labelled in any way in the text; each student statement can only be used once in a given question. However, multiple tags can be assigned to a single account, as evidenced from the the higher frequency of tag use compared to the total number of respondents.

Students were asked to try out the chatbot and then answer the following questions in Google Forms:

- Do you feel like you learned anything from it? Or that your students have learned something in it or with it?
- Did you feel drawn into the dialogue/learning process?
- Did you like the narrative approach of the chatbot? Was it believable to you?
- What would they do differently in the chatbot? What would you recommend to improve?
- How did you feel working with the chatbot?

Data collection took place between 27th February 2022 (the first response) and 28th March 2022 (the last response to the questionnaire). Responses to each question were exported to a text file, and this was then processed in the qualitative research application Taguette. As part of the data processing, we performed thematic analysis–individual questions from the questionnaire were further structured into themes so that they could be used in the discussion to create a set of recommendations for developing a chatbot to build information literacy among university students. In this respect, the study complements the more general recommendations [59] that draw from other areas.

The answers were formulated in Czech and Slovak, i.e., in the mother tongue of the respondents. The statements were also coded and processed in this language. It was only at the stage of text completion that the translation into English took place. This form also ensured the complete anonymity of the students. The individual students did not sign the assignments, so the researchers could not link individual responses to a specific person.

### 2.1. Description of Chatbot Francis

The research uses a design through research approach, which requires at least a brief description of the evaluated object. The chatbot is named “Francis”, and relies on a narrative approach (Francis has a memory problem, and students help him to remember what he has forgotten by searching for answers), and has already undergone two rounds of evaluation before the experiment. It was, therefore, a tested functional prototype.

**Figure 1:**
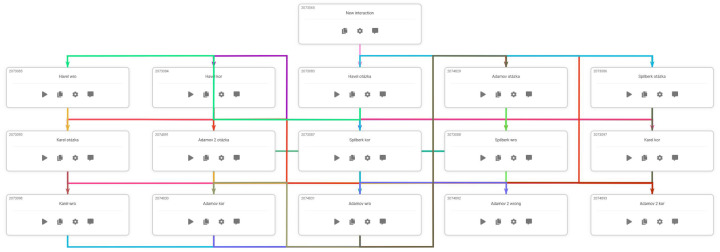
Schematic of the chatbot Francis in Snachbot.me.

The chatbot is made up of 16 interconnected frames in Snachbot.me. A diagram of the dialogue is shown in Figure 1. During the dialogue students are asked to answer the following questions:

- Do you know what month Václav Havel was born?
- Not far from Brno is said to be the ugliest town in the Czech Republic… Adamov. But what’s the name of the tower there?…they call it by one word in the vernacular…
- I’ve been thinking about one thing for a long time… I have a view out my window of a castle like this… Špilberk called it the prison of nations, but I’m talking about it again. Do you know who owns it today? (Please be precise!)
- And which tourist trail would you take from Adamov Square to that damn Alexandrovka?
- And something else slipped out… Charles I, the last Emperor of Austria–Hungary, was crowned King of Hungary on 30th December 1916, in Budapest. But what day was that? Monday, Tuesday or another day…I don’t know, can you tell me?

**Figure 2:**
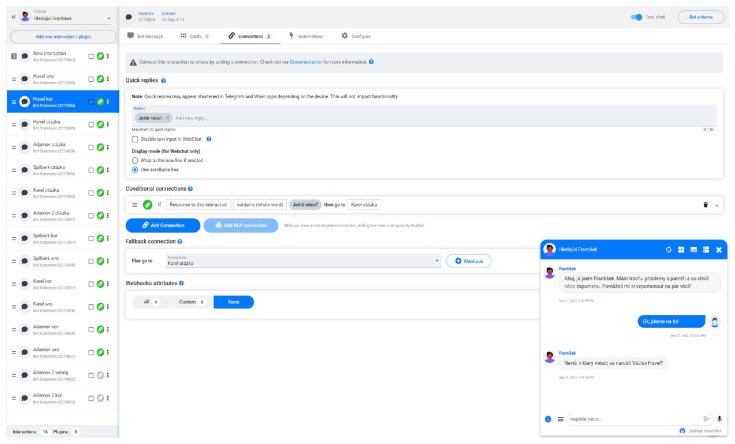
The Snachbot.me environment in which the chatbot Francis was created, and a sample of its dialogue.

The Snachbot.me application was chosen for its simplicity in creating a chatbot without using artificial intelligence. The development environment is shown in Figure 2.

It aims to develop competencies for searching for information, typically combining various tools (Google, Wikipedia, WolframAlpha, Mapy.CZ, Cadastre). This is a topic in which Information Studies and Library Science students should be experts and, at the same time, falls within the professional domain of their interest. If a student’s answer is not accepted, they will be given a hint as to where they might find the information.

The chatbot typically tries to find the search word in its common forms in the text string, which is only sometimes successful, as seen in the research results. A typical problem may be that some students at Czech universities do not speak Czech but Slovak. The correct answer must assume the Slovak shape for the first question, which was not the case (only Czech was considered and tested), which was a source of frustration for the Slovak students testing the chatbot.

### 2.2. Ethics of Research

In terms of research ethics, this research reflects the current critical debate over the ethics of scientific practice as described by Petousi and Sifaki [60]. Therefore, it seeks to maximise anonymity and protect users’ personal data, while ensuring sufficient scientific representativeness of the data. For this reason, the research does not make use of the logs from the actual dialogues in Snachbot.me, which were run with the chatbot by the students. Such an invasion of privacy would be unacceptable to research ethics. The research design critically reflected the ethical dimension of the research, especially in terms of data collection and processing. In the case of collection, this was primarily about setting up the anonymity of the research and making it voluntary; in the case of data processing, it was about procedures that do not allow anonymous data to be attributed to a specific user or a specific group of users.

Users worked with the chatbots independently, and no data was collected from their chats with the chatbot. Reflection was conducted voluntarily through Google Forms, which did not ask for the user’s name, email nor other identifiers. Thus, there was a separation of the dialogue data from the questionnaire data. In both cases, this was fully anonymous research in which the researcher could not identify an individual person. 

The questionnaire contained only optional questions (users could answer whatever questions they wanted). The socio-demographic questions were on age (interval delimited) and gender. These questions were also optional. By completing the questionnaire, users can use the data for research purposes. From the perspective of the research reader, the translation into English (the original answers were in Czech and Slovak) represents another step in the anonymisation process. The Helsinki Guidelines were followed in the research.

Thus, the research did not work with personal or sensitive data and did not require ethics committee approval.

## 3. Results

As already mentioned, a complete and comprehensive analysis of the responses is not crucial for the study. Still, we will observe their relation to pedagogical–psychological aspects that may be useful for the creators of chatbots without the use of artificial intelligence, and from which it will be possible to formulate individual recommendations or conclusions.

### 3.1. Do You Feel Like You Learned Anything from It? Or That Your Students Have Learned Something in It or with It?

The first question is aimed at justifying the use of the chatbot as an educational object. Looking at the available data in Table 1, we can say that the ratio of positive to negative feedback is about 3.6, with the caveat that if we include other tags (bugs, strange interactions, criticism of the chatbot’s length) in the negative feedback, the ratio will remain at a favourable 1.6. However, the objectives of our study are primarily qualitative.

Negative responses are often about problems with the design of a particular chatbot for a specific user: “No, he recommended links to tools I already know. I could have done without his ideas and found the answer to the questions without his help. Maybe it would be good for someone who is not knowledgeable in information retrieval”, “I already knew where and how to look for the information myself, so it wasn’t beneficial for me”, and, “Since the robot referred more to tools I already know, I didn’t learn much new in this regard”. This point implies the need for good research before a given chatbot is implemented in educational practice among the target group. Alternatively, one can work with differentiated tasks according to difficulty, if the user can choose them appropriately.

Regarding the responses in the category ‘Weird’, it can be said that they may be related to the limited possibilities of dialogue and its context in the technological solution: “I think it is good that Francis tells you where to look for the information he wanted to hear”, and, “The page I was redirected to, yes—it’s full of unique and most helpful information, the communication with Francis was strange, he was repetitive at first, it bothered me that he didn’t answer me in a very realistic way”. The design of the whole dialogue is crucial for real educational benefit and needs to be given enough attention. At the same time, it is interesting to note in this area that users explicitly mention the chatbot’s name, which happens in a relatively small number of responses (6 out of 74 in total—the exact proportion is not essential here, but rather serves a reminder of the fact that the atypicality of the communicative interaction leads to a higher need for respondents to refer to the chatbot by name).

The errors are characterised by the chatbot’s misunderstanding of the text input: “When he asks when Václav Havel was born, and I write 5.10.1936, he says that’s the wrong answer, I guess he has to write October”. This error is typically caused by the student not using spaces between the periods in the date. The perceived error may also be related to their ignorance: “He referred me to the site he wanted me to find the answer from, yet I searched for the answer on that site, but he still thought it was incorrect”, or related to their level of personal belief: “The chatbot did not accept the answers, but I knew what information to look for. However, the WolframAlpha recommendation was good, and I could finally try it out”. These examples show that in development, it is essential to test all possible answers carefully, to try to work with feedback in case of error and to strive for the best possible textual description of the situation under investigation.

For positive feedback, the need for students to point out a specific small piece of knowledge or experience that they take away is significant. This insight is crucial for the design of educational content, which needs to focus on (among other things) particular learning objectives that are graspable and clear to students, rather than more general competencies such as “I learned that I can use WolframAlpha more to find information” and “Working with cadastre”.

The second group of positive feedback is related to learning in general: “It’s beneficial; I feel like you can learn something with it”, “Yes, I think I have learned something, and I could use the chatbot to make my lessons more interesting”, and, “I think it’s great that I’ve tried looking for a direct query like this. This is enlightening for both potential students and me”. These examples show that the chatbot as an educational tool is crucial for users, as it is not conventional, and its form can appeal to students.

Some respondents worked in terms of linguistic means, with the chatbot being a real dialogue partner: “It’s a fun form of learning and by being involved in some “dialogue” one has more motivation to look up the information”, “The chatbot showed me some tools that I had not used or would not have thought to use to search for the kind of information that the chatbot was presenting”, and, “Francis recommends essential search tools that I already know, but I probably would not have thought to search for the owner by cadastre”. These references are valuable because they highlight the importance of authenticity, narrative and the language used in developing these entities.

### 3.2. Did you Feel Drawn into the Dialogue/Learning Process?

The second question was phrased as semi-open and tended towards shorter answers in a dual yes–no scheme. In terms of pull-ins (Table 2), we can see relatively balanced results, with the ratio of pull-ins to no pull-ins coming out at 0.97, or 1.27 when partial pull-ins are included. The semi-open formulation of the question leads to the fact that, although we have many responses (68), most do not contain any further explanation. Therefore, we will focus on the information relevant to the chatbot’s design in the results and their examples.

Students are drawn into the dialogue and often report sentences such as, “Francis has good reactions and believable, well-formed dialogue; I had no problem with the interaction. He even motivated me to do my research’”, and, “As soon as I got used to the style of question delivery, I got on nicely with the chatbot, and I feel like I kick-started my brain on the assignment/research/learning system. Mostly I enjoyed it, so it was a bit of a “learning by doing” experience for me, which I always really appreciate”, or even, “I guess you could say that, although I feel like the biggest idiot when talking to a bot”. The last statement is significant in that it shows an essential characteristic of the interaction, and perhaps its fundamental problem: communicating with a chatbot may be believable, but at the same time, it is strange, unusual, and not socially comfortable for a human to speak with a software entity through natural language. Authenticity, essential for immersion, is also a critical psychological limitation. These examples also illustrate that students understand interacting with a chatbot when they are drawn into a dialogue to be similar to how they would talk about a live teacher or tutor–it motivates them, gets their brain started, and talks to them. One student states, “Yes, even though the initial uncertainty, so much so that I was surprised myself”. The chatbot as a learning object is exciting and functional for a population segment that, in our research, seems to roughly correspond to half of the respondents. The other half is critical of it.

The critical comments are mostly very brief or general, yet we consider several of the responses necessary for designing other educational facilities of this kind. It may be a psychological barrier to communication with the machine: “I know that I am communicating with a chatbot, so there is a certain barrier that I am aware of”, or a combination of this factor and inaccurate responses to the answers: “Rather no, I could tell on Francis that he was a robot who had set some answers of his own, which were not always entirely relevant to mine”, and, “His way of communicating is too mechanical, and he could respond to words other than the correct ones”. Communicating with a chatbot can also be a source of some uncertainty: “I was sometimes confused when I got a completely different answer than I would have expected. This made me a bit uncomfortable”, and, “Unfortunately, it is recognisable that I am not communicating with a real person; I wanted to see how the chatbot would react when the storyline of the conversation did not go in the suggested direction, the chatbot responded with the same sentence over and over again”. These examples illustrate how essential it is to work with the dimension of the expected dialogue and the actions that can be predicted. The user has no “escape” options in the dialogue, which should lead the designer to require the active systematic building of a communication-safe environment, including, for example, the ability to advance the conversation further even if a question is not answered satisfactorily.

The last category mainly contains calls for prolonged dialogue: “My immersion was greatly reduced by the minimal interactivity of the bot”, and, “Easy, it was a relatively short experience”. Similarly, it also contains calls for more elaboration of the dialogue: “I think the discussion could have been more elaborate”. These points indicate that our expected parameter of a relatively short and quick dialogue can and should be modified to allow at least some users to have more sophisticated and extended interactions that lead to higher levels of immersion.

### 3.3. Did you Like the Narrative Approach of the Chatbot? Was It Believable to You?

In designing the chatbot, we drew on the principles that Wilcox and Wilcox (2013) described when they put narrative elements at the forefront of chatbot design. The results suggest that users are not uniform in this area: some would appreciate longer and more vivid dialogue of an almost novel kind, some value friendliness and authenticity, and others require error-free spelling or concise responses. A segment of users does not want to work with a chatbot because it is not a live human. At the same time, this attitude refers to the fact that they feel a certain authenticity of the dialogue, even if they say that the narrative approach was not desirable for them. The ratio of positive to negative responses (Table 3) in this question is around 1.3. If we include those who did not find the dialogue believable but still appreciated the narrative approach, the ratio is 1.5. In the table, we again list the tags used, and in the description of the examples, we will focus on practice recommendations.

Respondents comfortable interacting with the chatbot can be identified as, for example, “Within reason, yes. I could still tell I was talking to a robot and not a human, but the style of asking questions seemed quite natural”, “Yes, I liked the chatbot approach; it was believable and fascinating”, and, “Yes, I liked it. I wouldn’t say it was a robot, not a natural person”. All of these responses clearly show that the narrative approach leads users to think of the chatbot (despite knowing it is not human) as a human being. This finding is essential in applying chatbots to online education, as this can reduce feelings of loneliness and isolation. Another respondent’s statement echoes this: “I guess so. It’s a bit like Google assistant, except that with Francis, I have learned something extra–or rather, he didn’t spit out the answer right away but guided me to find it on my own”.

Specifically, respondents talked about the chatbot using less formal language and acting as a friend: “I would say yes. However, he came across as more of a friend than a teacher, but that is probably the intention of the chatbot”, or, “Yes, I found his friendly, informal approach quite comfortable and believable”. The dimension of linguistic appropriateness was intensely debated by the students and cannot be said to have been received in a purely positive way, however, this is something that did generate some discussion and reflection.

Among the negative comments, we came across an attitude criticising the fact that the chatbot is not human: “If one expects a chatbot, it’s normal; otherwise, it doesn’t seem very human”. As mentioned above, this chatbot is recognisable: although it is set up to use slang terms, it is evident that it has a clear storyline set, and it is not able to take the conversation in any other direction: “you could tell straight away that it was a chatbot, I don’t think anyone could mistake it for a human”.

The references to the coherence of the dialogue are significant: “Because of the disjointed nature of the individual questions and answers, it was not entirely believable to me, and this approach did not suit me”, “No. I found the given statements (chatbot questions) unnecessarily over-described; it was enough to ‘say’ briefly and simply what he was asking and what he needed to know”, and, “Halfway, it wasn’t very believable mainly because of the loss of the idea”. These comments suggest a recommendation to focus on clearer and tighter coherence of dialogue and possibly longer transition passages between interactions.

Within the analysis of the third category, the following statements can be mentioned as typical proxies: “It was nice that he could communicate naturally as if we were talking to a classmate and not to a robot or a subject who knows more”, (expressions like “HEY” by chatbot) “He was not believable because he made morphological mistakes in sentences”, and, “I think the approach was okay. Still, I don’t think he was believable”. Specifically, the statement, “You could tell it was ‘machined’. He came across as friendly, but at times it was so lecturing that I rolled my eyes”. This emphasises the authenticity of the response (leading to eye-rolling), at the same time as the social impression referring to a tone of voice and dialogue design that is authentic, while at the same time, an awareness that an honest conversation could probably look different.

### 3.4. What Would They Do Differently in the Chatbot, and What Would You Recommend to Improve?

Unlike the previous questions, this one is not directly about the chatbot and the chatbot experience, but rather focuses on student suggestions for improvement. Therefore, it does not make sense to construct positive and negative categories but instead to focus more on the individual areas of improvement that students addressed. The first and last categories in Table 4 are specific and do not make any practical recommendations. Therefore, we omit them in our work with student statements, although we still consider it essential to include them in the table.

Most of the recommendations and suggestions were related to improving the handling of responses. It is clear from the individual statements that students are not satisfied when the system does not sufficiently accept answers that they believe are correct or when it fails to guide the student to the correct answer: “I would improve the tolerance of answers towards the chatbot–not requiring a definite and very well-defined answer. I would also work on the morphology to make it more believable”, “More correct answers where it can be written in other ways”, and, “It would be good to improve the reception of answers in more extensive wording, or other falls. The chatbot would undoubtedly look more believable then. It would also be good if it had more possible solutions”. Some of these problems could be fixed by more rigorous and extensive testing.

Of note, some of the suggestions for improvement related to the absence of inserting the text answer directly: “I think in this case it would be useful to make some references from which the user could select the correct answer”, and, “Given the plethora of answer choices, I would prefer to give a multiple choice. The chatbot responded incorrectly to the answers”. The environment in which our particular chatbot is built allows for such interactions and represents a way of responding to a wrong/unrecognised answer where the second question does not expect text input but rather a button selection of the correct answer. Thus, there is a specific requirement to detect the concept of ‘don’t know’: “Could improve in response to other answers. For example, if I type ‘don’t know’, it might not answer ‘wrong’”.

The linguistic proposals for change touch on the areas already outlined above. Nevertheless, some new suggestions can be found: “Perhaps it might not be so overly positive (in another culture, it would be more appropriate)”. This statement refers to the Czech environment, which is much more damaging in terms of feedback than, for example, the US environment. Furthermore, there is a return to linguistic forms: “I would recommend leaving out phrases like ‘Hey dude, that’s some weird…’–it didn’t strike me as very credible, it bothered me”, and, “I would set the gender that the Chatbot should address others with to make it more believable” (Czech language contains gender distinction by verb endings and is thus manifested in almost all speech acts with the chatbot). There is also a repeated request for a more linguistically and literary-worthy finish: “Add a proper goodbye at the end to make it clear that it’s over”.

The changes we have identified as content-related concern the design of the whole dialogue: “I would change the composition of the chatbot questions to include questions traceable by the usual methods”, and, “I would improve the choice of questions or at least the way to ask them. I found many questions unrealistic or asked in a way one wouldn’t ask them”. Some users would welcome images in the chatbot: “I would add more photos to the pages mentioned”.

### 3.5. How Did You Feel Working with the Chatbot?

The last question often returns to the previous answers in the individual responses, which is understandable given their structure. Asking directly about feelings should help with the appropriate design of the learning object. It is clear from the reactions that it allowed for a more specific expression, often in a more open or direct form than in previous questions. As in previous questions, a single statement may have multiple tags (Table 5). Given the considerable embeddedness of the category of non-humanoid uncertainty, we do not calculate the ratio of positive to negative responses. Positive emotions can be expected to be related to the positive reactions made above.

We do not include the category ‘No emotion’ in the analysis; it is neutral and describes adequate communication not causing any particular emotion. A specific tag is ‘Arrogance’, in which students emphasise that they feel that the chatbot is not teaching them but instructing them, that it is being disrespectful or superior to them: “Like talking to a person who thinks a lot of himself and has to advise everyone”, “I felt like an idiot”, and, “Strange, terribly strange, but it was a happy experience, but I still prefer working with a living being. Francis seems arrogant; I didn’t like the work very much”. These responses have in common that the interaction is strongly perceived as human and simultaneously experienced as inappropriate and patronising. There is a need to work with this dimension in dialogue design. It does not have to be a priori about arrogance in the tone of voice but also about the respondent’s misunderstanding of what is being communicated during the dialogue.

This category is directly related to the one we have labelled ‘Non-humanoid uncertainty’. It is characterised by being ambivalent and accentuating the feeling of strangeness from dialogue with a machine (it is unnatural), and the sense of uncertainty (which again may be related to a combination of little experience with similar interactions and the chatbot’s lack of ability to respond appropriately to queries directed outside the dialogic framework). Examples of responses in this category might be “something between chatting with a human and a command line. I felt like I was in a study or experiment, wondering how the chatbot would respond, what responses it had pre-set, and whether it could consider the issue. The only thing that caught me off guard was that I didn’t know where my answers were stored”, “Interesting—a dialogue was being conducted, but at the same time, one was aware that one was not talking to a live person”, and, “A little strange to be writing to something I know is just a machine. If I didn’t know that, it would probably feel different, like a letter to someone you may not know personally, but you know the person will read it and express their opinions, feelings, etc”.

The above examples show that users perceive the chatbot as an inanimate person. Nevertheless, they place it in an epistemic framework that expects a person: “Since I knew that it was not a real person sitting on the other side, but a robot (even though it didn’t look like one), I felt a bit uneasy”, and, “Quite strange after realising that only a robot was communicating with me”. Many of the responses in this category were directed towards the problem of a specific difference between humans and machines experienced in the dialogue. The first statement is important from a design perspective, highlighting the need for ethical transparency, which is not easy to ensure in terms of the structure of dialogue, and may not be very influential when using specific tools. Feelings of confusion or uncertainty were frequent among respondents and were reflected on different levels. Together with the information from the ‘Arrogance’ tag, it can be said that we need to focus on building a safe environment for ethically transparent communication.

As such, there was a proportion of students who perceived the chatbot negatively, as in the previous questions, which was also reflected in their emotions: “Due to the limited interaction, almost a bit annoyed, there was no reason for me to try to write any meaningful response when I saw that it was only possible to get two responses for each answer, which is quite a shame for something that is probably meant to elicit a desire to interact”, “Inappropriately”, and, “Since the chatbot Francis’ expression was more limited, I didn’t feel very comfortable”. However, all of these responses suggest that a better design of the learning object could have made a significant positive difference to the whole situation.

The last category indicates a set of positive experiences: “I felt quite comfortable, I liked the variety of questions asked”, “The chatbot was fun to work with, even if its questions were tricky”. These questions relate strongly to the theme of working with a chatbot to the ability to structure one’s educational experience while interacting with this object. There are statements related to the simple statement of liking: “Pleasant”, “Pretty good”, or “I felt comfortable”. The conflict between comfort and uncertainty can be seen as a significant design challenge in developing this kind of educational object.

Another area is statements relating to the fact that the chatbot acted like a natural person: “Naturally. Like writing off an actual interviewer”, “Interesting–a dialogue was being conducted, but at the same time, the person was aware that they were not talking to a live person”, “To Francis in particular? Motivated”. It seems that, however limited it may be, the functionality of a chatbot without AI may lead to frustration or feelings of arrogance and insecurity, while at the same time, the literary form of the chatbot allows for such strong immersion in the dialogue that it feels like a living person, and so exact expectations are attached to it.

## 4. Discussion

Floridi [61,62] draws attention to the fact that modelling information interactions in the complex information environment we find ourselves in cannot be reduced to the interactions of people using tools, but that it is necessary to choose the path of the interactions of the informants. He understands an Inforg as an information agent that can change its state during an information exchange. Crucially for our research, this (partial) equality of human–chatbot interactions is a basic assumption with which students approach the interaction: parts of it come across as “weird” or “unusual”, but if we observe individual accounts (both critical and positive) we can say that users expect the interaction to be as close to human as possible. They project ethical and character traits onto the chatbot, manifested through tone of voice or preconceived interactions. Even a chatbot without artificial intelligence with minimal internal states represents a communication partner for students with whom they associate emotions and value expectations, which is considered a vital research result. The motivation with which we began the study, namely, the search for tools to reduce the social and psychological distance in online education, can be satiated by chatbots.

In research, the chatbot as an entity is located at the boundary of three domains of analysis: the informant, the educational object and the literary work. The use of chatbots (as a learning object) can be found in general frameworks following the development of e-learning [63], as well as in older texts from the beginning of the century. At the same time, we can note the growing popularity of this approach across cultures and cultural contexts [17,64,65]. We have mentioned the breadth of pedagogical uses ranging from fostering collaboration [43] to simulations of historical figures [42]. It is an essential educational tool that, according to student testimonies, makes sense both in AI-enabled systems and in situations where the possibilities of using AI are insufficient. All of the referenced studies say that the affective component of learning plays a vital role in the effectiveness and meaningfulness of implementing these objects. 

The chatbot may convey information, but its fundamental importance lies in removing the binary oppositional either/or schema and returning to how Dewey writes about emotions: “Joy, sorrow, hope, fear, anger, curiosity, are treated as if each in itself were a sort of entity that enters fully-made upon the scene, an entity that may last a long time or a short time, but whose duration, whose growth and career, is irrelevant to its nature. Emotions are qualities, when they are significant, of a complex experience that moves and changes” [66]. In other words, the goal of chatbots as educational objects (as follows from our findings) is to add an emotional dimension to educational entities and to the design of the whole academic environment, which is essential for the overall structuring of knowledge and the epistemic field in its breadth and depth. Chatbots are capable of fulfilling this function. We believe and, in light of the results presented above, we can rightly say that chatbots can help in the design of an educational experience that is meaningful, deep, and not associated with a purely cognitive reduction of learning from the perspective of the philosophy of education. We see this philosophical–pedagogical assumption as a fundamental reason for a more comprehensive implementation of chatbots.

In terms of our chatbot experiment on information literacy, it can be said that the use of chatbots in libraries is relatively common [67], albeit focused primarily on service delivery or broader information literacy issues [45,68]. In this respect, our experiment was relatively novel and provided a good rationale for why we adopted a design through research approach.

A specific problem that we encountered is the choice of tone of voice [69,70], where students felt that the chatbot was condescending, superior to them, could make them look stupid, etc. Di Gaetano et al. [71] point out this problem when they say that just setting the tone of voice is challenging. It is undeniably related to the limited ability of the chatbot to respond appropriately textually [15]. The chatbot enters education in a dialogic learning environment [72,73], however, it does not fulfil its ideals because it cannot respond to context without artificial intelligence. Although analysis of this problem can be encountered in a variety of settings [74] in the field of education, it is a very crucial topic that requires further research, an emphasis on prototyping, and a significant strengthening of testing chatbots before they are fully implemented educationally. In this regard, the chatbot must be a in specific literary form [75], playing the role of a non-linear interactive narrative [76] in which the student must change the flow and form of the dialogue through their decisions. If they fail to do so, they may be fazed by the flow of the interaction.

In the results section in the following chapter, we provide specific recommendations for designing learning objects, that is, chatbots without using AI systems. The individual points clearly show that a chatbot is a functional learning object that allows for the development of learning in a dialogical way. At the same time, a significant pitfall can be seen in this dialogue: tools without AI cannot work effectively with context, leading to user frustration and communication problems. Two facts emerge indirectly from the research that we would like to highlight:

- Users perceive the chatbot as an actor of information interaction, on which they place similar demands as on a human actor. Students perceive its strengths (e.g., ability to motivate, provide feedback, etc.) and weaknesses as manifestations of character. This finding has major philosophical [61,62] and design implications.
- Further research needs to focus on research into literary forms. The responses directly suggest that academic quality and the associated immersion, tone of voice, fluency and continuity of dialogue may be essential factors in improving the effectiveness and, most importantly, the experience and emotional design of working with a chatbot. The data suggest that treating the chatbot as a literary work is adequate.

The paper below identifies two to four key points for each question discussed in the results chapter that can be transferred to design practice, especially about the psychological and pedagogical aspects of working with a chatbot. Therefore, our recommendations do not focus on technical or educational pitfalls but mainly on the pedagogical–psychological aspect of chatbot design:

**Do you feel like you learned anything from it? Or that your students have learned something in it or with it?**

- The chatbot must articulate clear small learning objectives that are easily identifiable to the learner.
- The actual chatbot form can be fun for students.
- Chatbot functionality is essential for overall educational benefit.
- The chatbot can serve as a learning object, and students work with it in this way.

**Did you feel drawn into the dialogue/learning process?**

- The chatbot needs to be carefully tested before students work with it.
- The key is to look for strategies to build confidence and safety for the user, including the ability to skip parts of the dialogue.

**Did you like the narrative approach of the chatbot? Was it believable to you?**

- The choice of language and tone of voice is crucial to the believability of the dialogue and, at the same time, challenging to balance and perceive by the students.
- Some users find communicating in natural language with the machine challenging.
- Spelling errors should be avoided.
- Some users are inclined to welcome a more literary rather than concise conception of the chatbot.

**What would they do differently in the chatbot, and what would you recommend to improve?**

- Reacting to the term ‘I don’t know’.
- Careful testing of all answers.
- The repeating question does not expect a text input but a button selection of the correct answer.
- A clear conclusion is essential.
- It is necessary to think of the chatbot as a form of literary work.

**How did you feel working with the chatbot?**

- The quality of acceptance of correct answers is crucial for the quality of the chatbot.
- It is essential to test the tone of voice.
- It is essential to put the chatbot in a safe and transparent environment.
- It is essential to communicate the ethical aspects of working with chatbots (e.g., where interaction data is stored, who has access to it, etc.).

Some of the recommendations may be trivial for software developers, such as careful testing and absence of spelling error. In software development, chatbot developers can take inspiration from debugging chatbot “code”. On the other hand, our approach does not rely on the notion of chatbot creators as software engineers. However, by not requiring any particular technical skill to create, we are working more with the model of the developer of learning objects, for whom such practices may only partially be standard. For them, the trivial findings may be in the realm of clearly defining educational goals, which are understood here in the context of their clear communication to learners.

Huang et al. [53] discuss recommendations for chatbots directed towards language learning based on a review study. They identify an area of technological utility associated with requirements for the absence of error and ease of use. Furthermore, two dimensions of recommendations related to usefulness are present: pedagogical usefulness (which includes pedagogical activities related to providing quality information, responding to help, providing educational content, and working with learning scenarios) and social usefulness (interpersonal communication, open communication, and communication cohesion). Compared to our study focusing on information education, less emphasis can be seen on the development of competencies and the narrative level of the interaction between the student and the chatbot. The students in our research focus more on the psychological dimension than the analysis of Huang et al. [53] Technological simplicity in use is seen as essential by Vanichvasin [77].

The comparison with the study of Jin and Eastin [78] is fascinating: they recommend extroverted chatbot behaviour, whereas our respondents perceived this as more awkward or hostile. It would be helpful to investigate whether this difference is culturally determined, influenced by a particular implementation, or due to other (unknown) variables. According to Chew [79], the focus of development recommendations is on personalised recommendations, motivation, gamification, and emotional support. In our case, the first three items also appear in the research. In the last item, respondents who perceived a particular chatbot as arrogant can be identified, yet this again refers to the perceived importance of emotional support.

Wilkinson et al. [80] focused on chatbots recommending films and formulated two critical findings. Dialogue only needs to be of a reasonable length–providing all the information is not desirable as this leads to user fatigue. The second recommendation concerns the presence of a justification for a particular chatbot decision that users perceive as necessary, even though its form may be subject to debate. When analysing the responses more carefully, we can see an apparent inclination of users towards a narrative approach, which corresponds to a natural dialogue.

Wilcox and Wilcox [81] argue that chatbot development needs to incorporate a genuinely high-quality literary design, as with prose literature. The authors talk about the fact that the chatbot must have its own story, emotions, and typical reactions, while being technically perfect. Creating a narrative structure is essential, and a chatbot is a form of literary work. Stuij et al. [82] point out, however, that narrative may not be universally believable and that, on the contrary, it may present a psychological barrier for some users as they may perceive the chatbot as a technical object only. However, a substantial proportion of our respondents are much closer in their accounts to the notion of narrative chatbots conceived by Wilcox and Wilcox [81]. As the study by Abd-Alrazaq et al. [19] shows, narrativity or the use of a chatbot for therapeutic purposes, for example, need not be tied to artificial intelligence but depends more on the thoughtfulness of the entire dialogue. According to Chaves et al. [83], the appropriateness of linguistic resources is crucial for narrative design–the chatbot must speak appropriately, in language that is adequate to the situation and context. Adapting linguistic resources reduces credibility. Duncker [15] highlights the limits of practical dialogue and comprehension when interacting with a chatbot. In our research, when working with a chatbot without artificial intelligence, we can work with the objection to the absence of authentic dialogue by making the structure of the interaction more akin to a “theatrical play” with inappropriate language (on the part of the chatbot) than to an imitation of natural conversation.

## 5. Conclusions

On the one hand, working with chatbots without AI can be beneficial for students (as shown by their responses) and, at the same time, can provide essential insights into particular aspects of educational dialogue and learning process analysis for the design of systems using AI. Another advantage of chatbots without AI is the analysis of many students’ learning paths, which can serve for pedagogical research and evaluation of the educational process.

The research has shown that even a chatbot working based on dialogue frames without artificial intelligence can be a suitable and functional learning object that some users will like to work with, drawing them into the learning process (immersing) and motivating them to perform particular tasks. The research data shows that removing social and psychological barriers can make a chatbot valuable and functional. The rapid increase in the implementation of chatbots in educational practice correlates with the experience of the COVID-19 pandemic and the explicit requirement for e-learning to offer the development of cognitive goals and to work with emotion, experience, dialogue, and social interaction.

Whether it is systems without artificial intelligence or, for example, methods using GTP-3, their role lies precisely in the combination of cognitive and affective goals. The advantage of chatbots with clearly predefined tasks and dialogues is the possibility for the designer, as a professional teacher, to work through the logical structuring of selected parts of the explanation or the process of solving a specific task. In contrast, AI systems can focus more on authenticity and openness of communication as such. Students in this research emphasised the importance of knowledge goals when working with chatbots, which they often rated as key learning outcomes. The combination of motivation, fun and achieving knowledge-learning results presents one of the challenges we need to work with in object design. Regarding the actual recommendations for developers, these are summarised in list above, and we would like to present at least the most important ones here. During development, it is essential to work carefully with the prototype and to test it on the target group–this is a prerequisite for eliminating errors, considering all possible answers and choosing the optimal tone of voice. Nevertheless, it should be taken into account that a chatbot without AI will never be ideal, it will always contain errors, however, developers should be able to predict them and offer an environment in which the dialogue can continue even if there is a mistake. In many ways, students see the chatbot as a teacher, a source of motivation, support, feedback, and a guide through the learning process. This awareness should lead developers to respect high standards in their development.

It is unusual for students (at least in our research) to work with a chatbot, which leads them to demand both the overall quality of the dialogue (absence of errors, fluency, continuity of tasks), but also the need to build a safe environment–the possibility of working with the answer ‘I do not know’, the description of data processing, and other similar measures. We see the awareness that students need to have some certainty or sense of security in the dialogue as essential.

In terms of the design itself, it should be emphasised that the ability of the chatbot developer to work with a complex and adequate narrative is crucial. This creates an underlying emotional pre-understanding that strongly influences the immersion of the dialogue and the actual learning experience. An interesting finding of our research was that many users prefer more extended and more robust conversations to short questions with quick feedback.

Working with a chatbot without AI may have another interesting effect that we can see from the analysis of the results–some respondents are sceptical about communicating with technology and are unlikely to want to involve AI chatbots in their learning process (at least for now). The fact that there is a clear pedagogical intention of the teacher behind the chatbot may increase the credibility of such a learning object for a part of the respondents.

### Limitations of the Research

A limitation of the research is the relatively small sample size and especially the homogeneity of the respondents as students of information science and library science. In order to formulate broader principles for designing new chatbots, however, as the results of our research show, it would be appropriate to develop a new chatbot for each target group to educate them in an area that would be relevant to them. A limitation also lies in using a specific chatbot that would now–based on the data collected and subsequent refinement–be improved and would again provide a changed structure of feedback from students. In the future, research could be done with more chatbots. The second option is to incorporate the data from this research and the resulting recommendations into the chatbot and conduct another round testing and evaluation.

## Figures and Tables

**Table 1:** Educational possibilities of the chatbot Francis.

Tag	Description	Frequency
Beneficial	Responses included a positive emphasis on the educational benefit for the other or oneself.	54
Unprofitable	The response included believing that a chatbot is not a suitable educational object and that the individual (or anyone else) cannot learn anything from it.	15
Error	The response pointed out an error, real or perceived, that detracted from the satisfaction of the dialogic interaction.	6
Too short	The response stressed that the dialogue was too short to be able to clearly decide whether or not the user had learned something.	4
Weird	This category indicates some non-standard communication experience, usually negative, but not directly related to a denial of benefit or an explicit error.	8

**Table 2:** Engaging in the learning process with the chatbot Francis.

Tag	Description	Frequency
Drawn into dialogue	The response includes all positive responses in the questionnaire, including reactions such as “rather yes”. The aim is to collect positive responses to be drawn into the dialogue.	29
Partially drawn into the dialogue	The answers include a positive attitude towards the dialogue, but only after some other (satisfiable) condition has been met.	9
Not drawn into the dialogue	The response includes statements with a sense of lack of involvement in the dialogue, including “rather no”.	30

**Table 3:** Satisfaction with the narrative construction of the chatbot Francis.

Tag	Description	Frequency
The narrative approach suited me.	The answer includes all positive statements related to the narrative component of the dialogue, its believability and functionality. Words are retained even when the positive description contains a recommendation or reproach.	34
The narrative approach did not suit me.	Responses include a negative attitude towards dialogue and its believability. They are primarily directed against the very idea of a narrative approach applicable to chatbots without AI.	26
The narrative approach was not believable, but it suited me.	The answers highlight that a narrative approach can be a pleasant form of communication without necessarily being believable.	6

**Table 4:** Suggestions for improving the chatbot Francis.

Tag	Description	Frequency
Do not change anything.	Users in this category did not suggest any improvements and were fully satisfied.	8
Better interaction, response to answers	The responses form a broad group of suggestions for better working with acceptance and responding to the reactions. They are primarily based on the assumption that the chatbot does not accept all correct answers.	32
Language changes	Reactions associated with the suggestion to modify the language changes to the narrative, better storytelling, greater brevity, or removal of ungrammatical forms and spelling errors.	18
Content changes	Reactions focused on the chatbot’s content, difficulty, or development.	6
Rejecting chatbots	This category indicates responses with an explicit rejection of chatbots.	1

**Table 5:** Respondents’ feelings when interacting with the chatbot Francis.

Tag	Description	Frequency
Without emotion	The category includes statements that mention that the chatbot did not evoke any specific emotions and that they felt “normal”.	3
Arrogance	The category includes statements that directly label the chatbot as arrogant and lecturing or, conversely, the respondents as stupid.	8
Bad or unpleasant	The category refers to an otherwise unspecified area of experienced dislike or negative feelings.	11
Positive or drawn into dialogue	The intricately structured category includes positive statements focusing on working with chatbots–stating a positive experience, engaging in dialogue, fun or authenticity.	22
Non-humanoid uncertainty	The intricately structured ambivalent category includes statements about the unaccustomedness of working with a chatbot or communicating with a non-humanoid actor and elements related to feelings of insecurity.	22

## Data Availability

Data sharing not applicable. No new data were created or analyzed in this study. Data sharing is not applicable to this article.
